# Thermophilic cyanobacteria—exciting, yet challenging biotechnological chassis

**DOI:** 10.1007/s00253-024-13082-w

**Published:** 2024-03-21

**Authors:** Faiz Rasul, Dawei You, Ying Jiang, Xiangjian Liu, Maurycy Daroch

**Affiliations:** School of Environment and Energy, Peking University Shenzhen Graduate School, Shenzhen, 518055 China

**Keywords:** Thermophilic cyanobacteria, *Thermosynechococcus*, Photosystem, Phycobiliprotein, Chassis, Microalgae

## Abstract

**Abstract:**

Thermophilic cyanobacteria are prokaryotic photoautotrophic microorganisms capable of growth between 45 and 73 °C. They are typically found in hot springs where they serve as essential primary producers. Several key features make these robust photosynthetic microbes biotechnologically relevant. These are highly stable proteins and their complexes, the ability to actively transport and concentrate inorganic carbon and other nutrients, to serve as gene donors, microbial cell factories, and sources of bioactive metabolites. A thorough investigation of the recent progress in thermophilic cyanobacteria reveals a significant increase in the number of newly isolated and delineated organisms and wide application of thermophilic light-harvesting components in biohybrid devices. Yet despite these achievements, there are still deficiencies at the high-end of the biotechnological learning curve, notably in genetic engineering and gene editing. Thermostable proteins could be more widely employed, and an extensive pool of newly available genetic data could be better utilised. In this manuscript, we attempt to showcase the most important recent advances in thermophilic cyanobacterial biotechnology and provide an overview of the future direction of the field and challenges that need to be overcome before thermophilic cyanobacterial biotechnology can bridge the gap with highly advanced biotechnology of their mesophilic counterparts.

**Key points:**

*• Increased interest in all aspects of thermophilic cyanobacteria in recent years*

*• Light harvesting components remain the most biotechnologically relevant*

*• Lack of reliable molecular biology tools hinders further development of the chassis*

**Graphical Abstract:**

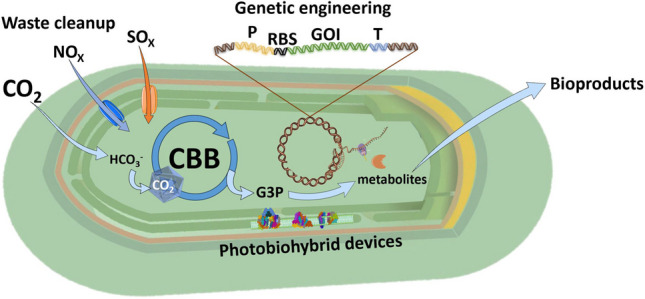

## Introduction to thermophilic cyanobacteria

Thermophilic cyanobacteria, usually found in hot springs, are prokaryotic microorganisms capable of growth between 45 and 73 °C and utilising oxygenic photosynthesis as a main source of energy and carbon. In this work, we define thermophilic cyanobacteria as members of cyanoprokaryota for which “part or all of their optimal growth temperature range is above 45 °C” according to a well-established definition by Castenholz (1969).

In addition to their high growth temperature, they sometimes exhibit polyextremophilic characteristics regarding high pH or elevated concentration of metal ions. These organisms are also essential primary producers of geothermal ecosystems that significantly contribute to carbon and nitrogen fixation and hot spring productivity. Together with other microorganisms, they usually form stratified microbial mats in thermal springs (Esteves-Ferreira et al. 2018; Kees et al. 2022).

**Fig. 1:**
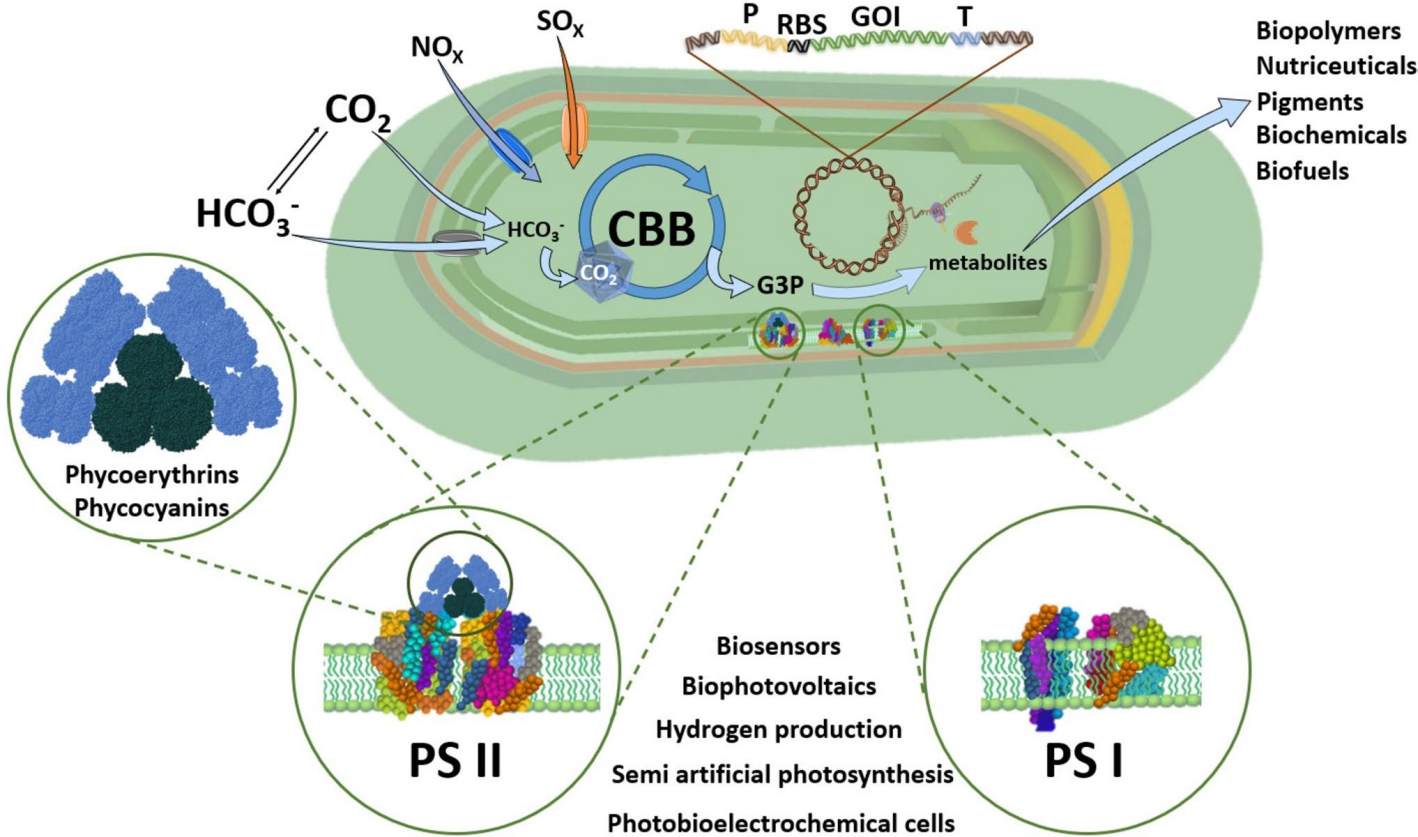
Overview of biotechnological applications of thermophilic cyanobacteria. P: promoter; RBS: Ribosome Binding Site; GOI: Gene of Interest; T: Terminator; PS II: Photosystem II; PS I: Photosystem I. The figure was partly generated using Servier Medical Art, provided by Servier, licensed under a Creative Commons Attribution 3.0 unported license

These robust photosynthetic microbes possess features that make them highly biotechnologically relevant (Fig. 1). Potentially useful natural products from thermophilic cyanobacteria include bioactive metabolites and polymers. They can also serve as thermostable gene donors for other organisms and as microbial cell factories for waste valorisation. The biggest advantages that the thermophilic cyanobacteria may have over their mesophilic counterparts are directly coupled to their high growth temperature. The most notable aspects include protection from microbial contamination and grazers that are incapable of withstanding high temperatures, biosynthesis of highly stable proteins and their complexes, and better prospects for transgene selection and biocontainment (Liang et al. 2019; Patel et al. 2019).

In recent years, there has been a renewed interest in these organisms leading to a significant expansion of knowledge about their taxa, properties, metabolism, and biotechnological potential (Saini et al. 2021). This is particularly evident in the recent surge in the delineation of novel thermophilic taxa such as the following *Thermoleptolyngbya* (Sciuto and Moro 2016), *Thermostichus* (Komárek et al. 2020), *Leptothermofonsia* (Tang et al. 2022b), *Trichothermofontia* (Tang et al. 2023), or *Thermocoleostomius* (Jiang et al. 2023). These are often combined with the collection and analysis of extensive genomic data about representative thermophilic isolates (Tang et al. 2022c). Simultaneously, widespread deployment of next-generation sequencing (NGS) technologies allowed further expansion of the sequence space among the thermosphere and depositing of numerous metagenome-assembled genomes (MAGs) from hot spring sequencing projects, opening more possibilities for biotechnological exploration of this resource. Despite the significant increase in the sequence information, thermophilic cyanobacteria remain relatively understudied in several aspects compared to their mesophilic counterparts, most notably in genetic engineering.

In this work, we attempt to synthesise the most important recent advances in thermophilic cyanobacterial biotechnology and provide an outlook into the future direction of the field. Such includes challenges that must be overcome before thermophilic cyanobacterial biotechnology can bridge the gap with more advanced biotechnology of their mesophilic counterparts.

## Biotechnology of thermophilic cyanobacteria: state of the art

### Thermophilic and thermotolerant cyanobacteria

#### Novel lineages and strains

To effectively implement cyanobacterial photosynthetic biomanufacturing technology, it is crucial to have access to high-light and temperature-tolerant strains that can be cultivated where insolation and temperatures are the highest and to utilise waste, high temperature sources of CO_2_. Thermophilic cyanobacteria possess characteristics that make them advantageous in these environments.

For many years, thermophilic cyanobacteria microbiology has been restricted to the isolates originating from the hot springs of Yellowstone National Park. Those organisms were extensively studied in their distribution, genomic variation and properties of individual isolates for nearly a century (Copeland 1936). Meanwhile, in the area of biotechnology works originating from Japan contributed the first complete genome sequence (Yasukazu et al. 2002) and transformation protocols (Iwai et al. 2004; Onai et al. 2004). In recent years, a growing number of thermophilic biological resources are being explored (Table 1). The increased knowledge pool was first evident in new strains of the unicellular strain *Thermosynecococcus* (Liang et al. 2019; Prondzinsky et al. 2021; Cheng et al. 2022) being described, presumably because of their abundance and ease of isolation. Now, an increasing number of thermophilic cyanobacteria are being isolated and characterised, sequenced, and developed as microbial cell factories. Several series of studies looking at novel thermophilic isolates proved to be successful in significantly expanding knowledge about new thermophilic cyanobacteria. A research line originating from western Sichuan hot springs sampling project (Tang et al. 2018) resulted in the delineation of three new genera (Tang et al. 2022b, 2023; Jiang et al. 2023) and the sequencing of several new genomes of novel species (Tang et al. 2021, 2022a). Elsewhere, a comprehensive study of cyanobacteria isolated from hot springs in Iceland, Poland, Greece, and Tajikistan resulted in thirteen new isolates and the delineation of two new genera (Jasser et al. 2022). India is also an important reservoir of thermal springs and related biodiversity. Nine hot springs in India’s northwestern Himalayas have been recently studied, resulting in describing three potential novel lineages of thermophilic cyanobacteria (Singh et al. 2018). Malaysian hot springs were also recently explored for the presence of novel organisms in their hot spring ecosystems and both unicellular *Thermosynechococcus* and *Leptolyngbya*-like forms exhibiting plant growth-promoting bioactivities have been identified (Wong et al. 2023). Another important recently described thermophilic strain is *Gloeomargarita ahousahtiae* exhibiting an optimal growth temperature of 45 °C (Bacchetta et al. 2023). The organism can form intracellular carbonate inclusions similar to some thermophilic strains of the genus *Thermosynechococcus*, but unlike these organisms, it prefers to use strontium and barium over calcium to form its intracellular deposits.

**Table 1:** Biotechnologically explored lineages and strains of cyanobacteria with thermophilic characteristics

Strain name	Phylum	Isolation source	Growth temp. (°C)	Accession number	Identified biotechnological potential	References
JSC-1	*Marcasia (Leptolyngbya)*	La Duke Hot Springs, Montana, USA	65	GCA_000733415	Potential for iron recycling through the formation of intracellular inclusions and extracellular ferrihydrite	(Brown et al. 2010)
BP-1	*Thermosynechococcus*	Hot spring, Beppu, Japan	55	GCA_000011345	Typical source of highly stable PS II and PS I complexes	(Yasukazu et al. 2002)
NIES 2134	*Thermosynechococcus*	Yunomine Hot Spring, Wakayama, Japan	57	GCA_003990665	Cellulose biosynthesis	(Kawano et al. 2011)
TA-1	*Thermosynechococcus*	Taian hot springs, Taiwan, China	50	GCA_017086385	Thermostable C-phycocyanin biosynthesis	(Leu et al. 2013; Cheng et al. 2022)
CL-1	*Thermosynechococcus*	Chin-Lun hot spring, Taiwan, China	62	GCA_008386235	Thermostable C-phycocyanin biosynthesis, Zn^2+^ bioremediation from wastewater	(Narindri Rara Winayu et al. 2021; Cheng et al. 2020)
PCC 6715	*Thermosynechococcaceae*	Hot spring, Yellowstone National Park, USA	55	GCA_002754935	Thermostable C-phycocyanin biosynthesis	(Liang et al. 2018; Tang et al. 2022c)
E542	*Thermosynechococcus*	Hot spring, Lotus lake, Sichuan, China	63	GCA_003555505	Bioremediation of flue gasses and fly ash, thermostable C-phycocyanin biosynthesis	(Liang et al. 2019)
Rupite	*Thermostichus*	Rupite Thermal Spring, Bulgaria	60	GCA_022848905	Highest growth temperature of all cyanobacteria	(Strunecký et al. 2019)
A121	*Leptodesmis*	Hot spring, Erdaoqiao, Sichuan, China	50	GCA_021379005	Antioxidant activity, nitrogen fixation capacity	(Tang et al. 2022a)
A174	*Thermocoleostomius*	Hot spring, Erdaoqiao, Sichuan, China	50	GCA_026802175	Capable of growth at high alkalinity, extensive CCM	(Jiang et al. 2023)
B231	*Trichothermofontia*	Hot spring, Zhonggu village, Sichuan, China	45	GCA_026240635	Cyanophycin biosynthesis, minimal CCM	(Tang et al. 2023)
E412	*Leptothermofonsia*	Hot spring, Lotus Lake, Sichuan, China	45	GCA_019891175	Thermostable C-phycoerythrin biosynthesis, far-red photosynthesis	(Tang et al. 2022b)
A183	*Thermoleptolyngbya*	Hot spring, Erdaoqiao, Sichuan, China	55	GCA_013177315	Nitrogen fixation ability, heavy metal ion adsorption	(Tang et al. 2021)
O-77	*Thermoleptolyngbya*	Hot spring, Kumamoto, Japan	60	GCA_001548395	Nitrogen fixation capacity	(Sciuto and Moro 2016)
PCC7942_HS199	*Synechococcus*	Evolved high-temperature phenotype	45	Mutant of NC_007604	Well-known microbial cell factory with an advanced genetic engineering toolkit	(Sun et al. 2023)

#### Increasing thermal performance of mesophilic strains

Apart from the isolation of novel thermophilic cyanobacteria, there is an increased interest in engineering and evolving thermal characteristics in model mesophilic chassis strains to take advantage of their more mature molecular biology toolkit. A variety of genetic engineering strategies have been explored to increase these organisms’ thermal resistance. Early works explored the overexpression of small heat shock proteins (Chaurasia and Apte 2009; Su et al. 2017). Whilst the overexpression effects were positive when compared to their respective wild types, none of the generated strains was capable of achieving continuous growth at or above 45 °C, borderline temperature to consider a strain thermophilic. These findings indicated the need for more sophisticated approaches in engineering thermal resistance in mesophilic cyanobacteria.

The discovery of a fast-growing and more thermostable strain of *Synechococcus elongatus*, UTEX 2973 (Yu et al. 2015), allowed for comparative analysis of this isolate against the near genetically identical model strain PCC 7942. The series of papers from various groups identified useful information about the genes involved in the strain’s enhanced thermostability. Among the different single nucleotide polymorphisms identified, a single mutation (C252Y) within *AtpA* gene, encoding the FoF1 ATP synthase subunit AtpA, stood out as the major contributor to temperature tolerance. To corroborate this mutation’s significance, it was introduced into the mesophilic model strain PCC 7942, resulting in a marked improvement in its stress tolerance and thermostability up to 43 °C (Lou et al. 2018). In-depth mechanistic investigations revealed that this mutation led to an increase in both the intracellular protein levels and enzymatic activity of FoF1 ATP synthase. It also had additional knock-on effects such as elevated intracellular *psbA* transcription, PS II/PS I ratio, linear electron transport rate, oxygen evolution rate, ATP levels, and glycogen accumulation under high-temperature stress conditions (Lou et al. 2018). This phenotype’s dominance over the wild-type PCC 7942 allowed for its markerless generation in just 4 days in a subsequent study (Zhang et al. 2021).

In addition to site-directed DNA alteration, random mutagenesis remains a potent technique for genetic improvement especially when the desired genotype, like thermal resistance, is fitness-bound and where specific genes requiring modification are unclear. The early works on employing multiple ultraviolet radiation (UV) and methyl methanesulphonate (MMS) mutagenesis resulted in boosting the thermal tolerance of a mixed mutagenized population of a model strain *Synechocystis* PCC 6803 by approximately 2 to 45 °C (Tillich et al. 2012). In the follow-up study focusing on monoclonal strains, enhanced thermal tolerance was validated. Whole-genome sequencing was performed, and the resulting data were compared to the wild-type genome to pinpoint the mutations responsible for these improved traits. The identified mutations were located within several genes: *clpC*, *pyrR*, *pilJ*, *pnp*, *pyk2*, *sigF*, *cya1*, and *nlpD*. Most optimal combinations of these changes allowed for growth at the maximal temperature of 45.8 °C, but the contribution of individual mutations was not elucidated (Tillich et al. 2014).

The most recent and arguably the most successful approach is the combination of an evolutionary route with site-directed engineering of DNA repair and recombination machinery in *Synechococcus* PCC 7942. The developed hypermutation system allows for rapid generation and functional screening of millions of mutants, and it has been employed to identify high-light high-temperature mutants of this model strain (Sun et al. 2023). By using this effective system, *Synechococcus* point mutants were isolated that displayed enhanced tolerance to high light and high-temperature stress. The resultant strain HS 199 can grow at 45 °C with a light tolerance of up to 2500 μmol photons/m^2^/s, unattainable for any rationally constructed variant of PCC 7942. The genomic basis of this phenotype was identified, with one specific point mutation found in the upstream non-coding region of a gene-encoding shikimate kinase. This mutation led to increased gene expression in the identified strain and had a remodelling effect on the photosynthetic chain and metabolic network (Sun et al. 2023).

All the attempts described above indicate that there is a plethora of genes such as alterations in photosystems, oxygen and ROS tolerance, and ATP generation contributing to the thermostability. They also show that thermostability is a complex phenomenon that requires the action of numerous genes and is unlikely to be improved with simple genetic manipulation to bring the optimal growth temperatures of engineered strains above 45 °C and closer to those of native thermophilic cyanobacteria. In the future, targeted efforts can be useful to combat these processes in a combinatorial fashion to expand the strain’s ability to overcome stresses related to temperature and light.

### Biotechnological applications for thermophilic cyanobacteria and their components

#### Thermostable photosystems

Historically, the interest in thermophilic cyanobacteria in the context of biology and biotechnology stems from the robustness of their light-harvesting apparatus (Zilliges and Dau 2016; Zhang and Reisner 2020). This made *Thermosynechococcus elongatus* (nowadays *Thermosynechococcus vestitus*) and *T. vulcanus* model organisms for the structural studies of photosynthesis, and especially photosystems II and I (PS II and PS I). The high stability of these complexes allowed for the elucidation of the structures of both PS I (Jordan et al. 2001; Çoruh et al. 2021) and PS II (Zouni et al. 2001; Kato et al. 2021), initially with X-ray crystallography and more recently with cryo-electron microscopy. In recent years, additional techniques complemented by quantum chemical calculations have been employed for the mechanistic and structural studies of the Kok-Joliot cycle in Thermosynechococcus (Cox et al. 2020; Lubitz et al. 2023). Techniques include the following: X-ray diffraction with free-electron lasers (XFEL), electron paramagnetic resonance (EPR) and related double resonance techniques (electron-nuclear double resonance—ENDOR, and electron double resonance-detected nuclear magnetic resonance—EDNMR). These advances cemented the position of the organism as a model for mechanistic studies of photosynthesis.

The superior stability of those two complexes compared with their mesophilic counterparts and plants has later been adopted for biotechnological solutions like the construction of biohybrid devices such as biophotoelectrodes and biophotovoltaics (Kargul et al. 2012; Musazade et al. 2018) and remains along with the photosystems of extremophilic red algae of the order Cyanidiales (e.g. *Cyanidioschyzon* sp., *Galdieria* sp.) as the most promising solutions for light harvesting needed by biohybrid devices. The very high stability of those complexes remains crucial to this day both in structural biology and in biotechnology. In structural biology, there has been a significant focus in recent years on dissecting the structures of remaining components of the light-harvesting systems such as NAD(P)H dehydrogenase complex (Laughlin et al. 2019), photosystem assembly intermediates (Xiao et al. 2021; Lambertz et al. 2022) and in the structural features of photosystems deficient in certain structural components (Huang et al. 2021; Boussac et al. 2022; Nakajima et al. 2022) and containing critical point mutants (Takegawa et al. 2019; Xiao et al. 2020). Albeit sparingly, some structural studies also focused on other thermophilic cyanobacteria that do not belong to the genus *Thermosynechococcus*. Here, the work on the cryo-EM structure of tetrameric PS I from *Chroococcidiopsis* (Semchonok et al. 2022) and early structural studies of the cytochrome b6f from *Mastigocladus laminosus* (Kurisu et al. 2003) could be highlighted.

The interest in using photosystems, originally isolated from spinach in biohybrid devices started with their immobilisation on platinum electrodes (Agostiano et al. 1983). Since those early works, and especially after the protocols of their efficient isolation and purification from *Thermosynechococcus* have been well established (Boussac et al. 2004; Kern et al. 2005; El-Mohsnawy et al. 2010), the emphasis of the work on biohybrid devices has moved beyond the biological aspect of the photosystems and towards their integration with increasingly sophisticated materials. Whilst both photosystems responsible for light harvesting in natural photosynthesis are currently used for the construction of biohybrid devices, either as separate light-harvesting complexes or as purified thylakoid membranes that contain a full complement of membrane-associated proteins, their application is slightly different and corresponds to their original biological functions.

Photosystem II (PS II) is the first complex in the sequence transforming the light energy into activated electron carriers, widely known as the light phase of photosynthesis. The unique feature of this complex is its ability to split the substrate water molecules into molecular oxygen, electrons and protons at a rate of 100 molecules per second (Kato et al. 2013), opening an array of possibilities for the photobiosynthesis of fuels or chemicals, generation of electricity and remediation of pollutants (Zhang and Reisner 2020; Xuan and Li 2021). The first application of thermophilic PS II from *T. elongatus* immobilised on a graphite electrode was used for the construction of biosensors for the detection of herbicides in water (Koblížek et al. 2002). The subsequent improvement to the design came through the utilisation of His-tagged PS II and its effective directional immobilisation on nitriloacetic acid–modified gold electrodes and nanoparticles (Maly et al. 2005; Badura et al. 2006; Terasaki et al. 2008; Noji et al. 2011). Those early designs typically allowed for low photocurrent densities below 2.5 μA cm^−2^ (Zhang and Reisner 2020). Further enhancement of electron transfer efficiency was possible with the incorporation of osmium-modified redox polymer hydrogel that had a dual function of immobilization matrix and electron acceptor. This approach allowed for an over tenfold increase in current density and significant improvement in stability (Badura et al. 2008). The development of second-generation, three-dimensional, mesoporous electrodes made of electrically conductive metal oxide like ITO (indium-tin oxide) allowed for better, often oriented, absorption of PS II on the surface of the material and direct transfer of electrons to the electrode (Kato et al. 2012, 2013). Although this approach resulted in no major improvements in photocurrent generation due to insufficient pore sizes of the material, it was an important step for the development of the third-generation electrodes made of hierarchically structured inverse opal mesoITO (IO-mesoITO) (Zhang and Reisner 2020). The third-generation electrodes are characterised by all the benefits of mesoporous conductive ITO electrodes enhanced with the presence of chambers and channels that allow better photosystem anchoring and substrate/product diffusion. These features allowed the devices based on *T. elongatus* PS II and IO-mesoITO electrodes enriched with redox polymer to generate photocurrent densities approaching 1 mA cm^−2^, 400 times higher than early devices (Sokol et al. 2016; Zhang and Reisner 2020). Electrode designs based on IO-mesoITO have been successfully improved with hierarchical assembly (Fang et al. 2019), and used for the synthesis of hydrogen (Mersch et al. 2015; Sokol et al. 2018). The cross-utilisation of IO-mesoITO, osmium-modified redox polymer hydrogel, and various phycobilisome (PBS) components allowed for further improvement of the device’s quantum efficiency (Hartmann et al. 2020).

Photosystem I (PS I) complex isolated from thermophilic cyanobacteria of genus *Thermosynechococcus* are widely known for their remarkable stability exceeding three months (Iwuchukwu et al. 2010). This, combined with the greatest in biology reduction potential of − 1.2 V and internal quantum efficiency approaching 100%, makes them the most popular components for biohybrid solar devices. Due to a similar light-harvesting function as that of PS II, the design of electrodes and assembly of biological and abiotic components follows a similar path. Early designs relied on gold (Terasaki et al. 2009), platinum (Iwuchukwu et al. 2010), subsequently, ITO, TiO_2_ (Kondo et al. 2014), and graphene (Feifel et al. 2015b, 2015a) became increasingly popular. Simultaneously, the directional orientation of immobilised PS I through electrostatic alignment or application of osmium-modified redox polymer hydrogels allowed for more efficient electron transfer. Unlike the PS II which is capable of water-splitting reaction and extracting electrons from an abundant source, native PS I relies on the extraction of electrons from cytochrome C_6_. When in vitro systems are applied, the designs frequently mimic this solution by providing a recombinant version of this electron shuttle (Kölsch et al. 2018) or providing an alternative, often sacrificial electron donor such as ascorbate (Nagakawa et al. 2019; Torabi et al. 2022). Similarly, to the works on PS II, researchers are actively interested in increasing the light-harvesting spectrum of the complex. Whilst native phycobilisomes have been utilised in PS II (Hartmann et al. 2020), synthetic dyes were often applied in PS I. Most notable examples include Atto 590 (Gordiichuk et al. 2016) and Lumogen Red (Nagakawa et al. 2019) both targeting the green gap of photosystem I. An alternative and successful method to enhance the performance of isolated thermosynechococcal PS I is its coupling to metal nanostructures in the form of nanoparticles (Ashraf et al. 2016), nanodiscs (Pamu et al. 2021), or silver islands (Czechowski et al. 2014). The resultant enhancement of fluorescence in the range of several to several 100-fold can be attributed to the plasmonic effects of these nanostructures coupled with a fluorophore (Kowalska et al. 2020).

Finally, several examples included the application of both photosystems in biohybrid devices to mimic the Z-scheme of photosynthesis. The combination of the two photosystems mitigates key drawbacks of both complexes, i.e., relatively low energy of electrons excited by PS II and the need for sacrificial electron donor by PS I. The early coupling works included the application of artificial electron shuttles such as 2,6-dichlorophenolindophenol (Kopnov et al. 2011) to permit semi-artificial photosynthesis in solution, separating the two photosystems embedded in osmium-modified redox polymer into two separate photoelectrodes (Kothe et al. 2013) or overlaying and crosslinking two isolated cyanobacterial photosystems onto a single electrode (Yehezkeli et al. 2013; Efrati et al. 2013). Whilst similar works are still attempted (Zhao et al. 2019), the challenges associated with the precise directional assembly of the biocomponents within the device and charge recombination, and the long-term instability of PS II, resulted in the shift towards attempts to reproduce the Z-scheme from its native components in vitro into fully abiotic or hybrid devices in which at least one of the light-harvesting components is synthetic.

#### Thermostable photopigments

Thermophilic cyanobacteria, capable of light harvesting at elevated temperatures, have a significant advantage over mesophilic strains considering the biosynthesis of robust biopigments. In recent years, there have been significant efforts to unveil the overall structure of the PBS complex including allophycocyanin cores and peripheral phycocyanin rods isolated from *Thermosynechococcus vulcanus* NIES-2134 (Kawakami et al. 2021, 2022).

On the applicative front, several strains of unicellular thermophilic cyanobacteria, i.e., NIES 2133, NIES 2134, PCC 6715, and PKUAC-SCTE-E542 were explored as an efficient source of thermostable blue pigment C-phycocyanin. Results have shown that PCC 6715 had higher growth rates and its C-phycocyanin maintained 90% stability after 5-h incubation at 50 °C and maintained good long-term stability (Liang et al. 2018). Further studies investigated the optimal light colour and regime conditions for the growth and phycobilin production of PCC6715 in the tubular photobioreactor (Klepacz-Smółka et al. 2020). Elsewhere, the C-phycocyanin (C-PC) extracted from *T. elongatus* TA-1 demonstrated sustained integrity and functionality across a broad temperature spectrum (4–60 °C), maintaining 65.65% activity at the upper limit of 60 °C. It also has shown stability under varying pH conditions, ranging from 4 to 9 (Leu et al. 2013). Allophycocyanin (APC) β-subunit from *T. elongatus* BP-1 also demonstrated the capacity for long-term stability (Puzorjov and McCormick 2020). The APC α-subunit in *T. elongatus* BP-1 was more stable than those of mesophilic strains, showing only a 5% reduction in fluorescence when incubating at 65 °C for 1 h (Chen et al. 2016). Furthermore, heterologous expression of phycocyanin operon from *T. elongatus* (*cpcBACD*) in *Synechocystis* strain has been successful in yielding thermostable product at 112 ± 1 mg g^−1^ DW and improved the growth rates (Puzorjov et al. 2021). These works indicate that thermosynechococcal phycobiliproteins could replace Spirulina phycocyanin as a more stable photopigment.

The identification of chlorophyll d (Chen et al. 2010) and chlorophyll f (Chen et al. 2010) and the discovery of far-red light–acclimating cyanobacteria were important steps in our understanding of the limits of cyanobacterial photosynthesis (Gan and Bryant 2015). The identification of thermophilic filamentous cyanobacterium, *Marsacia* (*Leptolyngbya*) JSC-1 (Brown et al. 2010), capable of chlorophyll f biosynthesis and far-red photosynthesis followed (Gan et al. 2014). Detailed studies of this phenomenon led to the discovery of far-red absorbing allophycocyanin, its recent detailed structural studies (Gisriel et al. 2023), elucidation of far-red cyanobacteriochromes (Moreno et al. 2020), and heterologous expression of thermophilic far-red–absorbing phycobiliproteins of *Thermostichus* A1643 and *Leptolyngbya* JSC-1 in *Escherichia coli* (Soulier et al. 2020) and *Synechococcus* sp. PCC 7002 (Soulier et al. 2022), respectively indicating multiple mechanisms of far-red adaptation.

#### Biopolymers from thermophilic cyanobacteria

Cyanobacteria have been long considered as a source of valuable bioproducts, and this also applies to their thermophilic representatives. In addition to the above-discussed photopigments, cyanobacterial biopolymers are also the subject of active research. Cyanophycin, a polymer composed of aspartic acid and arginine, synthesized by a respective synthetase (Kwiatos and Steinbüchel 2021), is one of the substances frequently synthesized by thermophilic cyanobacteria. The biopolymer is a nitrogen storage compound, which may be particularly valuable in oligotrophic environments such as thermal springs. Initial work on thermophilic producers of this compound was done on unicellular strain MA19 (Hai et al. 2002) and was followed by a study in model organism *Thermosynechococcus* BP-1 (Arai and Kino 2008). More recently, other thermophilic members of cyanobacteria, such as *Chlorogloeopsis fritschii*, have been explored as producer organisms for the biopolymer (Jyoti et al. 2019). The gene encoding the enzyme catalysing the biosynthesis, cyanophycin synthase *CphA* is a near ubiquitous feature in most genomes of non-nitrogen fixing *Thermosynechococcus* and frequently found in the most thermophilic of all cyanobacteria *Thermostichus* (unpublished results), further suggesting a correlation between nutrient scarcity in the hot springs and mechanisms of their storage. Another important biopolymer identified in thermophilic cyanobacteria is cellulose. It has been identified that *Thermosynechococcus vulcanus* NIES 2134 propensity for cellular aggregation at blue light and low temperature is resultant of its ability to synthesize and secrete cellulose (Kawano et al. 2011). In follow-up studies, expression profiles, deletion, and overexpression mutants of the strain were studied, and cyclic diguanylate was identified as a key regulatory molecule for cellulose synthesis in *Thermosynechococcus* (Maeda et al. 2018; Enomoto et al. 2018).

#### Bioremediation with thermophilic cyanobacteria

High optimal temperature of growth, the presence of multiple CO_2_ and HCO_3_^−^ transporters (Tang et al. 2022c) and good biomass productivities of several thermophilic strains (Hsueh et al. 2007; Bergmann and Trösch 2016) make these organisms promising candidates for the valorization of carbon and nutrients from the flue gasses. Despite these advantages, only one study proposed the utilization of thermophilic cyanobacteria, of the genus *Thermosynechococcus*, as microbial cell factories for carbon sequestration and valorization (Liang et al. 2019). Whilst the study had its impact restricted by a poor growth rate of the studied strain there are plenty of representatives of the genus with significantly higher growth rates that could be developed into microbial cell factories. In addition to carbon sequestration from the flue gasses, efforts have been made in the utilization of *Thermosynechococcus* CL-1 strain for the nitrogen and carbon reduction in swine wastewater (Narindri Rara Winayu et al. 2021). The same strain has been found as a promising organism for the removal of estrogens from wastewater, further indicating the metabolic potential of thermophilic cyanobacteria (Chang et al. 2021). Meanwhile, thermophilic representatives of *Phormidium* sp. have been successfully tested as biosorbents for phenol removal from aqueous solutions (Karatay et al. 2017) and other hot spring strains for heavy metal absorption (Seda Şen et al. 2022; Al-Qahtani et al. 2023).

## Genetic engineering and practical considerations

Despite several reports on the genetic engineering of thermophilic cyanobacteria (Table 2), the molecular toolkit for thermal strains lags significantly behind that of their mesophilic counterparts, where major alterations of cellular metabolism, deployment of genome editing protocols (Wendt et al. 2016; Knoot et al. 2020) and integration of multiple cassettes spanning tens of kilobases were achieved (Zheng et al. 2023).

**Table 2:** Successful attempts in engineering thermophilic cyanobacteria

Name	Promoter	GOI	Terminator	Ab resistance	Ori	Integrative	Remarks	Host strain	Ref
pC*km*_Te_-based TS1_Te_, TS2_Te_, TS3_Te_, TS4_Te_	PcpcC	*kanNT*	T4 terminator	Thermostable kanamycin (*km*_*Te*_)	P15A	Yes	Natural transformation/electroporation	*T. elongatus BP1*	(Onai et al. 2004)
pUC18 and pBSCM13^+^	Plac	*PsaF, PsaL, PsaK*	Not mentioned	Kanamycin/chloramphenicol/spectinomycin	pBR322	Yes	Electroporation/conjugation	*T. elongatus*	(Mühlenhoff and Chauvat 1996)
pT7Blue	Plac	*aadA*	Not mentioned	Streptomycin	pBR322	Yes	Electroporation	*T. elongatus BP1*	(Iwai et al. 2004)
His-*psb28*-pUC18	Plac	*Psb28*	NOS	Chloramphenicol	pBR322	Yes	Natural transformation	*T. vulcanus*	(Xiao et al. 2021)
pUC18-* psbV*	Plac	*psbV*	NOS	Streptomycin	pBR322	Yes	Natural transformation	*T. vulcanus*	(Xiao et al. 2020; Huang et al. 2021)
BP1-pKA	PnirA	*kdc*-*adh*	rbcS	Kanamycin	pMB1	Yes	Electroporation	*T. elongatus BP1*	(Sacko et al. 2020)
BP1-BY20	PnirA	*eyfp*-*rol*	rbcS	Tetracycline	pMB1	Yes	Electroporation	*T. elongatus BP1*	(Sacko et al. 2020)
pUC43-H-psbA3	PpsbA3	*psbA3*	TpsbA3	Gentamycin	pBR322	No	Electroporation	*T. elongatus*	(Takegawa et al. 2019)
pEdNblA	PsbAII	*aadA*	rrnB T1	Spectinomycin	pUC19	Yes	Natural transformation	*Thermosynechococcus sp.* E542	(Liang et al. 2019)
pETS1	PsbAII	*aadA*	rrnB T1	Spectinomycin	pUC19	Yes	Natural transformation	*Thermosynechococcus sp.* E542	(Liang et al. 2019)
pETS2	PsbAII	*aadA*	rrnB T1	Spectinomycin	pUC19	Yes	Natural transformation	*Thermosynechococcus sp.* E542	(Liang et al. 2019)
pLJD31	PpsbA*	*cscB*	rrnB T1	Kanamycin	RSF1010	No	Conjugation	*Thermosynechococcus sp.* E542	(Cui et al. 2022)
pETS1	PsbAII	*aadA*	rrnB T1	Spectinomycin	pBR322	Yes	Natural transformation	*Thermosynechococcus sp.* E542	(Riaz et al. 2021)

In principle, there are two approaches for the genetic engineering of cyanobacteria: utilisation of self-replicative vectors or genomic integration, and thermophilic cyanobacteria are no exception. Whilst the former may result in significant variability in gene expression and does not allow for deletion-based functional studies, the latter offers greater stability and site-specificity. When exogenous genes surrounded by homology arms enter the cyanobacteria, genomic integration occurs mainly through homologous recombination. There are two main methods of homologous recombination in the cyanobacteria. The first is that cyanobacteria can naturally absorb foreign plasmids or linear DNA for genome integration without the need for auxiliary tools; the second requires the generation of DNA strand breaks, for example using the CRISPR/Cas9 system. When it comes to the introduction of exogenous DNA into cyanobacteria, three approaches are typically used: natural transformation, conjugation, and electroporation. The first, natural transformation, utilises the natural ability of many cyanobacteria to absorb plasmid or linear DNA from the environment. This is the simplest and most convenient way to introduce foreign DNA into cyanobacteria including thermophilic strains (Onai et al. 2004). Secondly, there is conjugative transfer that relies on *E. coli* as a carrier of conjugative plasmids, such as RP4 or constructs based on RSF1010 origin of replication (Cui et al. 2022) and subsequently delivers DNA through conjugation. The method is most established in UTEX 2973 that cannot be transformed using natural transformation protocol (Yu et al. 2015). However, it has been recently adapted to *Thermosynechococcus* E542 to generate a sucrose-secreting strain that could provide other members of the bacterial community with photosynthetically produced carbon source (Cui et al. 2022). The high optimal growth temperature of this cyanobacterium allows for its relatively easy purification from the *E. coli* carrier strain that is unable to grow at 50 °C. Finally, electroporation that uses a high-intensity electric field to instantly increase the permeability of the cell membrane, allowing for the absorption of exogenous molecules, appears to be the most universal approach that can be used for thermophilic strains (Iwai et al. 2004).

The elevated optimal growth temperature of *Thermosynechococcus* compared to other cyanobacteria prompted its utilization as a biocontainment organism that could prevent transgene escape into the natural environment (Yamaoka et al. 1978). More detailed studies revealed that whilst the organism is indeed a poor grower at temperatures 30 °C and below, but it is not killed in those mesophilic temperatures (Sacko et al. 2020). Consequently, it is not a sufficient biocontainment mechanism, and more sophisticated approaches should be employed. Its high optimal temperature for growth could possibly be used as a protection mechanism against eukaryotic grazers that are detrimental to the many mesophilic cyanobacteria and microalgae (Liang et al. 2019).

## Outlook

Critical evaluation of the recent progress in thermophilic cyanobacteria reveals that despite a significant increase in the number of newly isolated and delineated organisms, there are still significant gaps in knowledge and application of these organisms at the high end of the biotechnological learning curve, as well as the many seemingly obvious benefits of these organisms that remain relatively unexplored. The bulk of most recent findings could be attributed to the field of taxonomy genomics and metagenomics. Yet, despite an abundance of sequence data on thermophilic strains in the form of MAGs and complete genome sequences, this pool of sequences did not translate into their biotechnological applications, with the exception of expression of thermophilic photopigments in mesophilic hosts (Puzorjov et al. 2021). There are still areas to be explored where thermostable cyanobacterial proteins could have significant benefits over their mesophilic counterparts, and it could become one of the active areas of research for the near future, notable examples include enzymes, chaperons, and light harvesting components.

Whilst photosystems of thermophilic cyanobacteria remain the largest biotechnological application of these organisms, there is a glaring lack of any biological advancement in the field of photosystem genetic improvement and most important, repair. It appears that the entire focus of the research community is directed toward the material component of biohybrid devices, especially electrodes and to lesser extent mediators, and not on the biological one. Further biological improvement of photosystem stability, especially that of PS II, could bring additional benefits to the material aspect of these devices. Furthermore, in analysing the construction of those biodevices, there is an excessive focus on solutions that are not compatible with photosystem-based light-harvesting components. The biggest advantage of PS-based light harvesting over synthetic counterparts is the device’s ease of production, low cost, potential scalability, and biodegradability. Therefore, combining them with osmium-based polymers that are based on toxic, critical raw materials is not particularly compatible. In our opinion, increased efforts should be directed at unveiling and employing mechanisms of photosystem replacement and in situ repair to generate better synergy.

Elsewhere, whilst numerous manuscripts have been published on the valorisation of CO_2_ to high-value products by mesophilic cyanobacteria, little progress has been made with thermophilic counterparts. Utilisation of thermophilic strains could be especially valuable when industrial sources of CO_2_ such as hot temperature flue gasses are proposed as primary carbon source (Klepacz-Smolka et al. 2024). Production of engineered products in thermophilic chassis could complement and expand the native metabolic capacity of organisms such as biosynthesis of biopolymers (cyanophycin, cellulose) or photopigments paving the way for industrial CO_2_ biorefinery-based valorisation. This method of cyanobacteria cultivation, providing that satisfactory growth rates are obtained, could result in better overall process efficiencies as strains grown at elevated temperatures could be protected against eukaryotic grazers incapable of surviving in those high temperatures.

Many of these applications are still restricted by immature genetic engineering toolkits even for the relatively well-known strains belonging to the genus *Thermosynechococcus*. Even those relatively simple organisms lack reliable genetic modules and advanced genome manipulation methods. In cyanobacteria in general, but especially in *Thermosynechococcus*, it is more difficult to achieve specific integration due to less effective homologous recombination machinery (unpublished data) and attempts to develop protocols of obtaining miniploid cells are not as successful as those employed for mesophilic model strains (Riaz et al. 2021, 2022). Moreover, in the absence of established genome editing tools based on CRISPR/Cas9 methodology, presumably due to the high toxicity of nucleases (unpublished data) and potential thermal instability of mesophilic proteins, specific genome engineering of thermophilic cyanobacteria remains a tedious task. These issues are further compounded by poor availability of genetic modules and thermal instability of selection markers and many antibiotics. Some of those issues could be partially alleviated by the utilisation of inducible promoters or control elements, but these are even less available than in mesophilic counterparts. One of the approaches that may be attractive and specific to thermophilic organisms is the cis-level expression control based on RNA thermometers (RNAT) that have been described in *Thermosynechococcus* before (Cimdins et al. 2014).

These enduring problems with the efficiency of genetic engineering in thermophilic cyanobacteria prompted many groups to engineer and evolve better thermal characteristics in well-established mesophilic model strains such as *Synechococcus* PCC 7942. Several approaches have been tested but arguably, due to numerous pathways on how improved thermal characteristics of the strain can be achieved, the evolutionary approach based on hyper-mutation strain followed by population resequencing and reverse engineering is the most promising of all. Time will tell if those efforts could result in the mesophilic strains with characteristics approaching those of naturally occurring thermophilic strains, but the direction is clearly worth observing.

To summarise, improvements of the genetic toolkit of naturally occurring thermophiles, evolving new thermophilic variants of well-known model chassis and biological improvement of photosystems derived from thermophilic strains could be highlighted as essential lines of research for the future.

## Data Availability

The original contributions presented in this review are included in the article. Further inquiries can be directed to the corresponding author.
